# Computed Tomography Verified Prevalence of Incisional Hernia 1 Year Postoperatively after Colorectal Cancer Resection

**DOI:** 10.1177/1457496920976053

**Published:** 2020-12-16

**Authors:** Niklas Karlsson, Sophia Zackrisson, Pamela Buchwald

**Affiliations:** Department of Surgery, Skåne University Hospital, Lund University, Lund, Sweden; Department of Translational Medicine/Diagnostic Radiology, Lund University, Lund, Sweden; Department of Surgery, Skåne University Hospital, Lund University, Lund, Sweden; Department of Translational Medicine/Diagnostic Radiology, Lund University, Lund, Sweden; Department of Surgery, Skåne University Hospital Malmö, Lund University, Lund, SE-205 02, Sweden

**Keywords:** Incisional hernia, computed tomography, colorectal cancer, surgery, minimally invasive surgery

## Abstract

**Background and objective:**

Incisional hernia is a frequent negative outcome after open and minimal invasive surgery of colorectal cancer. This study aimed to determine computed tomography–verified incisional hernia prevalence 1-year post colorectal cancer surgical resection in patients sutured with standardized small stich 4:1 technique, identify risk factors for incisional hernia and assess to what extent incisional hernia required surgical correction.

**Methods:**

All patients subjected to resectional colorectal cancer surgery during 2012–2016 at Skåne University Hospital were identified in the Swedish Colorectal Cancer Registry. The 1-year follow-up computed tomography was re-evaluated to establish the presence of incisional hernia. Clinical data were collected from Swedish Colorectal Cancer Registry and the patients’ medical charts were reviewed. Non-parametric tests and binary logistic regression analysis were used for statistical analysis.

**Results:**

In total, 1744 tumors were identified resulting in 1231 patients meeting the inclusion criteria. In total, 25.9% (n = 319) had incisional hernia at the 1-year follow-up computed tomography and 13.2% (n = 162) of the colorectal cancer resections were minimal invasive surgery, and there was non-significant incisional hernia prevalence difference between open and minimal invasive surgery. However, for converted and non-converted minimal invasive surgery, the incisional hernia frequencies were 43.9% (n = 18) and 24.1% (n = 39), respectively (p = 0.012). Significant risk factors for incisional hernia were body mass index, wound rupture, and procedure time. During the follow-up time, 14.1% (n = 45) needed incisional hernia corrective surgery.

**Conclusions:**

Incisional hernia after colorectal cancer surgery is common despite standardized small stich 4:1 closure, but few incisional hernias are surgically corrected. Incisional hernia is equally frequent after open surgery and minimal invasive surgery. However, the risk of incisional hernia is considerably higher after minimal invasive surgery conversion.

## Introduction

Incisional hernia (IH) is a common complication following abdominal surgery including surgery for colorectal cancer (CRC). The IH incidence after open CRC surgery has been reported to be between 10% and 32% and similar in minimal invasive surgery (MIS), that is, laparoscopic- or robotic-assisted CRC resections^1–3^. IH develops over time where studies suggest that 54% and 75% have occurred within 1 and 2 years postoperatively, respectively^4,5^. Advanced age, obesity, superficial surgical infection (SSI), wound dehiscence, and previous laparotomies have been proposed risk factors for IH^5–7^. IH can be detected during clinical examination or with imaging techniques such as ultrasound, computed tomography (CT), and magnetic resonance imaging (MRI). Abdominal CT has best sensitivity and most reproducible results regarding IH diagnosis^1,8^. However, it is not indisputable how to analyze different modalities as the variance of detection rate and interobserver agreement between studies have been reported to be significant^1^. The IH incidence is generally higher when using imaging techniques compared to clinical examination. Furthermore, all diagnosed IHs are not clinically relevant in terms of symptoms.

During the last decade, a new standardized small stich 4:1 closure of the abdomen has been introduced to reduce IH rates^9–11^.

The primary aim of this study was to determine the IH prevalence after open and MIS of CRC in patients that have been sutured with the standardized small stich 4:1 closure. The secondary aims were to identify risk factors and to resolve how often IHs were surgically corrected.

## Material and Methods

A retrospective review of patients who had undergone open or MIS of CRC during 1 January 2012 to 31 December 2016 at Skåne University Hospital Malmö (SUS), Sweden, with a follow-up thoracoabdominal CT examination 12 ± 3 months postoperatively were included. All patients were followed until 1 April 2019. Clinical variables were extracted from the Swedish Colorectal Cancer Registry (SCRCR), namely, age at diagnosis, gender, date of surgery, weight, length, body mass index (BMI), tumor localization, neoadjuvant treatment, American Society of Anesthesiologists (ASA) classification, type of surgical procedure, surgical approach, whether the surgical procedure was acute or elective, duration of surgery, perioperative bleeding, if the patient got a stoma or not (regardless of permanent or temporary), tumor staging according to histopathology, and postoperative complications. In MIS cases, the registrations, if the procedure was laparoscopic- or robot-assisted and if the procedure had been converted, were noted. All incisions including MIS extraction site incisions were midline.

The standardized small stich 4:1 technique was implemented almost 10 years ago at SUS. 2.0 PDS suture is used and suture remnants and length of fascia defect are measured, and a ratio is calculated.

All CT examinations at 1-year ± 3 months follow-up were reviewed using Picture Archiving and Communication System (PACS IDS7) Sectra AB, Linköping, Sweden. IH was defined as “an abnormal protrusion of the contents of the abdominal cavity or of preperitoneal fat through a defect of weakness in the abdominal wall at the surgical scar”^12^. The follow-up CT was compared to the preoperative CT to determine if the IH had evolved post CRC resection. Only patients without previous IH were included. All CT reviews were done by a senior radiologist. In case of an IH, medical charts were reviewed to determine if a surgical corrective IH procedure was carried out and if so with which technique. This follow-up was performed 1 April 2019. The study was granted ethical approval by the Swedish Ethical Review Authority. Strengthening the Reporting of Observational studies in Epidemiology (STROBE) guidance for reporting of observational studies was followed.

### Statistical Analysis

Categorical risk factors were assessed for correlation with IH using Pearson’s χ^2^-test. For continuous normal distributed variables, that is, age and BMI, Student’s t-test was used, while Mann–Whitney U-test was used for non-normal variables, that is, operating time and bleeding. For variables showing a p value < 0.2, a logistic regression analysis was carried out to investigate potential associations between risk factors and development of IH. The same procedure was performed in the IH-positive groups to assess factors affecting the chance of having IH surgery. p values < 0.05 were considered significant. All calculations were performed using SPSS version 26.

## Results

### Study Population

**Fig. 1.**
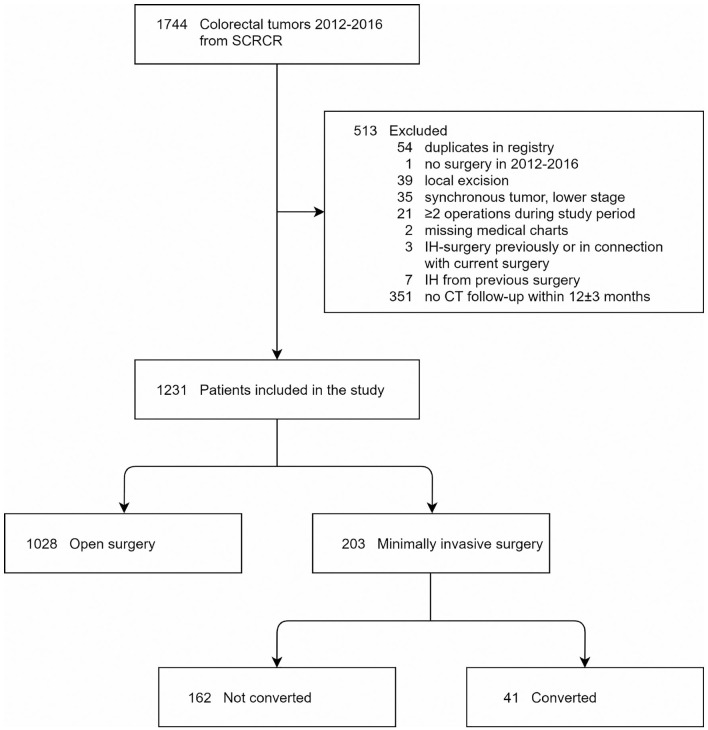
Patient flowchart of this study. SCRCR: Swedish colorectal cancer registry.

In total, 1744 resections for CRCs were registered at SUS in SCRCR during 2012–2016. In total, 513 patients were excluded most commonly due to no follow-up CT within 12 ± 3 months, surgery not matching the inclusion criteria, for example, transanal endoscopic microsurgery or polypectomy and synchronous tumors. After exclusion, 1231 patients remained of whom 1028 and 203 patients were subjected to open CRC surgery and MIS, respectively, the latter group was divided into converted or not (Fig. 1).

### Clinical Characteristics and Exclusion Analysis

Clinical characteristics of the study population and the excluded patients are shown in Supplemental Table 1. Noticeable differences between the groups were age, ASA classification, and acute CRC surgery (p < 0.001).

### Study Outcomes

In total, 319 (25.9%) of the patients had developed IH at the follow-up CT. There was no significant difference in IH prevalence between open CRC and MIS non-converted (p = 0.566), but a significant difference in IH prevalence between converted and the non-converted MIS 43.9% and 24.1%, respectively (p = 0.012). Risk factors for IH development were BMI (p < 0.001), operation time (p = 0.021), colon cancer surgery (p = 0.001), wound dehiscence (p = 0.002), stoma (p = 0.025), reoperation (p = 0.013), and high T-stage (p = 0.049) (Tables 1 and 2). For the potential risk factors, defined as p value < 0.2, a binary logistic regression analysis was performed showing remaining risk factors, expressed in odds ratios (OR): BMI (1.09; p = 0.018) and wound rupture (3.94; p < 0.001; Table 2 and 3).

**Table 1.** Summary of patients and incisional hernia characteristics.

Variable	Group	All	IH positive	IH negative	p value
		n = 1231	n = 319	n = 912	IH positive versus IH negative
Age (years)		68.8 (10.8)	69.4 (10.2)	68.6 (11.0)	0.245^a^
Gender	Male	656 (53.3)	160 (50.2)	496 (54.4)	0.193
	Female	575 (46.7)	159 (49.8)	416 (45.6)	
BMI (kg/m^2^)		25.8 (4.9)	27.2 (6.3)	25.3 (4.2)	**<0.001** ^a^
Resected area	Colon	767 (62.4)	223 (69.9)	544 (59.7)	**0.001**
	Rectum	463 (37.6)	96 (30.1)	367 (40.3)	
ASA classification	1–2	805 (65.9)	196 (62.0)	609 (67.2)	0.094
	3–4	417 (34.1)	120 (38.0)	297 (32.8)	
Operation type	Elective	1086 (88.4)	278 (87.1)	808 (88.9)	0.403
	Acute	142 (11.6)	41 (12.9)	101 (11.1)	
Bleeding (mL)		250 (100, 500)	200 (100, 200)	250 (100, 500)	0.144^b^
Operation time (min)		232 (175, 336)	220 (166, 322)	236 (180, 342)	**0.021** ^b^
T-stage	T0–T2	226 (18.5)	47 (14.8)	179 (19.8)	**0.049**
	T3–T4	998 (81.5)	271 (85.2)	727 (80.2)	
N-stage	N0	630 (51.6)	162 (50.9)	468 (51.9)	0.773
	N1–N2	590 (48.4)	156 (49.1)	434 (48.1)	
Neoadjuvant therapy	Yes	165 (13.4)	32 (10.0)	133 (14.6)	**0.038**
	No	1063 (86.6)	287 (90.0)	776 (85.4)	
Preoperative radiation	Yes	319 (26.0)	71 (22.3)	248 (27.3)	0.078
	No	909 (74.0)	248 (77.7)	661 (72.7)	
Stoma	Yes	549 (45.0)	125 (39.6)	424 (46.9)	**0.025**
	No	672 (55.0)	191 (60.4)	481 (53.1)	
Wound infection	Yes	66 (5.4)	17 (5.3)	49 (5.4)	0.976
	No	1165 (94.6)	302 (94.7)	863 (94.6)	
Wound rupture	Yes	32 (2.6)	16 (5.0)	16 (1.8)	**0.002**
	No	1199 (97.4)	303 (95.0)	896 (98.2)	
Reoperation	Yes	20 (1.6)	10 (3.1)	10 (1.1)	**0.013**
	No	1211 (98.4)	309 (96.9)	902 (98.9)	
Surgical technique	Open	1069 (86.8)	280 (87.8)	789 (86.5)	0.566
	MIS	162 (13.2)	39 (12.2)	123 (13.5)	
Procedure by area	Right colon	408 (34.0)	115 (36.4)	293 (33.1)	**0.014**
	Left colon	246 (20.5)	78 (24.7)	168 (19.0)	
	Rectum	547 (45.5)	123 (38.9)	424 (47.9)	

IH: incisional hernia; BMI: body mass index; MIS: minimal invasive surgery.

Numbers in parentheses are percentages. Unless otherwise stated, p values were calculated using Pearson’s χ^2^-test. Significance level used is 0.05 and the significant results are marked in bold.

a Student’s t-test.

b Mann–Whitney U-test.

**Table 2.** Summary of IH positive subgroup with respect to IH surgery.

Variable	Group	IH positive	IH surgery	no IH surgery	p value
		n = 319	n = 45	n = 274	IH surgery versus no IH surgery
Age (years)		69.4 (10.2)	68.1 (8.0)	69.6 (10.5)	0.280^a^
Gender	Male	160 (50.2)	31 (68.9)	129 (47.1)	**0.007**
	Female	159 (49.8)	14 (31.1)	145 (52.9)	
BMI (kg/m^2^)		27.2 (6.3)	27.4 (3.8)	27.1 (6.6)	0.733^a^
Resected area	Colon	223 (69.9)	28 (62.2)	195 (71.2)	0.225
	Rectum	96 (30.1)	17 (37.8)	79 (28.8)	
ASA classification	1–2	196 (62.0)	38 (84.4)	158 (58.3)	**0.001**
	3–4	120 (38.0)	7 (15.6)	113 (41.7)	
Operation type	Elective	278 (87.1)	42 (93.3)	236 (86.1)	0.181
	Acute	41 (12.9)	3 (6.7)	38 (13.9)	
Bleeding (mL)		200 (100, 200)	250 (100, 500)	200 (100, 450)	0.708^b^
Operation time (min)		220 (166, 322)	259 (166, 379)	216 (166, 313)	0.259^b^
T-stage	T0–T2	47 (14.8)	8 (17.8)	39 (14.3)	0.541
	T3–T4	271 (85.2)	37 (82.2)	234 (85.7)	
N-stage	N0	162 (50.9)	29 (64.4)	133 (48.7)	0.051
	N1–N2	156 (49.1)	16 (35.6)	140 (51.3)	
Neoadjuvant therapy	Yes	32 (10.0)	7 (15.6)	25 (9.1)	0.183
	No	287 (90.0)	38 (84.4)	249 (90.9)	
Preoperative radiation	Yes	71 (22.3)	13 (28.9)	58 (21.2)	0.249
	No	248 (77.7)	32 (71.1)	216 (78.8)	
Stoma	Yes	125 (39.6)	19 (42.2)	106(39,1)	0.693
	No	191 (60.4)	26 (57.8)	165 (60.9)	
Wound infection	Yes	17 (5.3)	2 (4.4)	15 (5.5)	0.776
	No	302 (94.7)	43 (95.6)	259 (94.5)	
Wound rupture	Yes	16 (5.0)	9 (20.0)	7 (2.6)	**<0.001**
	No	303 (95.0)	36 (80.0)	267 (97.4)	
Reoperation	Yes	10 (3.1)	6 (13.3)	4 (1.5)	**<0.001**
	No	309 (96.9)	39 (86.7)	270 (98.5)	
Surgical technique	Open	280 (87.8)	34 (75.6)	246 (89.9)	**0.007**
	MIS	39 (12.2)	11 (24.4)	28 (10.2)	
Procedure by area	Right colon	115 (36.4)	15 (33.3)	100 (36.9)	0.714
	Left colon	78 (24.7)	10 (22.2)	68 (25.1)	
	Rectum	123 (38.9)	20 (44.4)	103 (38.0)	

IH: incisional hernia; BMI: body mass index; MIS: minimal invasive surgery.

Numbers in parentheses are percentages. Unless otherwise stated, p values were calculated using Pearson’s χ^2^-test. Significance level used is 0.05 and the significant results are marked in bold.

a Student’s t-test.

b Mann–Whitney U-test.

**Table 3.** Binary logistic regression analysis of variables with p value < 0.2 for all versus incisional hernia (IH)-positive groups and for IH positive versus IH surgery groups.

Group	Variable	p value	OR	OR, 95% CI
IH positive versus IH negative	Gender (male)	0.302	1.161	0.875–1.540
	BMI	**<0.001**	**1.090**	**1.056–1.125**
	Resected area (colon)	0.164	0.669	0.379–1.179
	ASA classification	0.474	1.113	0.830–1.494
	Bleeding	0.194	1.000	0.999–1.000
	Operation time	0.741	1.000	0.999–1.001
	T-stage	0.142	1.336	0.907–1.968
	Neoadjuvant therapy (no)	0.142	0.690	0.421–1.131
	Preoperative radiation (no)	0.090	1.612	0.929–2.796
	Stoma (no)	0.740	0.926	0.588–1.459
	Wound rupture (yes)	**0.018**	**3.909**	**1.265–12.082**
	Reoperation (no)	0.823	0.853	0.210–3.453
IH surgery versus no IH surgery	Gender (male)	**0.020**	**0.408**	**0.192–0.870**
	ASA classification	**0.001**	**0.204**	**0.077–0.541**
	Operation type (elective)	0.670	0.746	0.195–2.864
	Surgical technique (MIS)	**0.013**	**3.167**	**1.278** 7.850
	N-stage	**0.032**	**0.440**	**0.207–0.933**
	Neoadjuvant therapy (no)	0.278	1.814	0.619 5.316
	Wound rupture (no)	**0.017**	**8.766**	**1.463** 52.510
	Reoperation (no)	0.771	1.384	0.155 12.395

OR: odds ratio; CI: confidence interval; BMI: body mass index; MIS: minimal invasive surgery.

The reference used for nominal variables is shown in parentheses. Significance level used is 0.05 and significant results are marked in bold.

In total, 45 (14.1%) patients underwent corrective surgery for IH until 1 April 2019 with a median follow-up time of 534 days. In a majority of the IH cases (55.6%) the sublay technique (retro muscular) was used, while the second most frequent method was an inlay technique (15.6%). Furthermore, for the patients who developed IH, the probability of having corrective surgery was analyzed. The probability of having corrective surgery for an IH after non-converted laparoscopic surgery was 28.2%, while the corresponding number for open surgery was 12.1% (p = 0.007). The identified factors affecting the risk of having corrective surgery for an IH were male gender (p = 0.007), low ASA classification (p = 0.001), wound rupture (p < 0.001), reoperation (p < 0.001), and MIS (p = 0.007). Binary logistic regression analysis showed gender (OR = 0.4; p = 0.0020), ASA classification (OR = 0.2; p = 0.001), N-stage (OR = 0.4; p = 0.032), MIS (OR = 3.2; p = 0.013), and wound rupture (OR = 8.7; p = 0.017) to be factors affecting the chance of having corrective surgery (Tables 1 and 2).

## Discussion

This study shows IH in one-fourth of patients 1 year postoperatively indicating that IH is a common complication to CRC surgery in a modern cohort. There was no significant difference in IH prevalence between open and MIS surgery but significantly higher if the surgery was converted into open surgery. Wound rupture was a strong risk factor for IH. Only a minority of patients (14.1%) underwent corrective surgery. Thus, IH is still a major complication after CRC surgery that needs further attention even in the 21st century.

The prevalence of IH 1 year postoperatively found in this study is high. A systematic review including 14,618 patients found an IH rate of 12.8% at an average follow-up time of 23.7 months, though the IH range of the included studies varied from 0% to 35.6% (6). However, most patients were diagnosed by clinical examination (67%) and a minority by radiological methods (9%)^6^. Other studies have reported IH incidences of 10%–18%^7,13,14^. CT has been demonstrated to have both high sensitivity and high specificity^1,15^ but a limited interobserver agreement^8^. Although only one radiologist was involved in this study, many small IHs were identified which may explain the high IH frequency. IH develops over time and some small IH may become clinically relevant later^4,5^. It is reasonable to expect that small defects that present early, possibly caused by inadequate wound healing, after prolonged tissue stress gradually increases the risk of developing a clinically significant IH.

The standardized small stich 4:1 technique, where the incision is closed suture usage four times the length of the incision, has been suggested to reduce IH rates^9^. Despite the standardized small 4:1 technique of the midline incision used in this study the IH detection rate was high which may not only be attributed to the CT diagnosis. Primary reinforcement of the incision with mesh lowers the IH incidence but is not appealing in surgery that includes colon or rectal resections^16^. There are indications that a reinforcement suture may be of interest. An ongoing clinical trial called Rein4CeTo1 has the aim to compare IH incidence after open CRC surgery when two different stitching techniques are used, either standardized small stitch suture or a reinforced tension line suture in addition to the standardized small stitch suture^17^.

Several studies indicate that there is no difference between open surgery and MIS in IH incidence^3,7,13^. This study is in accordance with previous findings but identified MIS conversion to be risk factor. There are different reasons to MIS conversions which may be confounders explaining the higher IH prevalence in this group. Nevertheless, the conversion itself may be the cause, since the initial procedure plan was abandoned hence a new situation aroused with non-optimal conditions.

Risk factors for developing IH have been reported in other studies. Previous suggested risk factors are high BMI, male gender, procedure time > 3 h and wound complications^7,18,19^. In this study, high BMI and wound rupture were significant risk factors. Another study which also emanated from SCRCR showed somewhat different results^19^. However, the follow-up time was 5 years and the presence of IH was extracted from the National Patient Registry (NPR) in Sweden, that is, only diagnosed IHs were included resulting in IH incidence of 5.3%. Since that study exclusively was based on registry data and no CT examinations or medical charts were reviewed, only IHs severe enough to be reported to the NPR were included. Furthermore, all laparoscopic procedures were excluded thus our results appears valid given the few surgical IH corrections.

One study reports the 5-year probability of having IH surgery to 4.1% for open surgery and 3.2% for laparoscopic surgery^20^. A large systematic review estimates the probability of having corrective surgery for IH after open surgery to 5.2%^6^. This study shows a higher probability to undergo corrective surgery. No obvious explanation has been found but some of the difference could be related to the large number excluded from the study due to missing follow-up CTs as this shifts the population in the less comorbid direction and hence patients more fit for surgery.

Limitations of this study consist of the large number of exclusions, which partly has been accounted for through a solid exclusion analysis. Since the study was based on SCRCR, only variables present in the registry were taken into consideration and hernia size, loss of domain, hernia-related quality of life, and cancer recurrence were not accounted for. Furthermore, complications, such as anastomotic leakage and wound rupture, are underreported in SCRCR, but the anastomotic leak rate at Skåne University Hospital during the study period was 5%. The reported wound rupture prevalence in our data set was just above 5% and although most of patients developed IH the numbers were too small to be significant. Strengths are that the study population reflects a routine CRC service with standard praxis including abdominal closure where everyone is trained in the standardized small stich 4:1 technique. Moreover, all CT images have been reviewed by one radiologist.

Further studies are needed to reach conclusions on how to minimize the risk of developing IH and to understand the relevance of small IH found at the 1-year follow-up. Future studies on surgical techniques are essential.

In conclusion, IH after CRC surgery is common despite 4:1 fascia closure, but few IHs are corrected surgically. IH is equally frequent after open surgery and MIS. However, the risk of having IH is considerably higher after MIS conversion.

## Supplemental Material

sj-pdf-1-sjs-10.1177_1457496920976053 – Supplemental material for Computed Tomography Verified Prevalence of Incisional Hernia 1 Year Postoperatively after Colorectal Cancer Resection Click here for additional data file. Supplemental material, sj-pdf-1-sjs-10.1177_1457496920976053 for Computed Tomography Verified Prevalence of Incisional Hernia 1 Year Postoperatively after Colorectal Cancer Resection by Niklas Karlsson, Sophia Zackrisson and Pamela Buchwald in Scandinavian Journal of Surgery
