# Supplementary material for: Computed Tomography Verified Prevalence of Incisional Hernia 1 Year Postoperatively after Colorectal Cancer Resection
Source: Scand J Surg. 2020 Dec 16;110(3):373–9. doi: 10.1177/1457496920976053 (PMC8551438; doi:10.1177/1457496920976053)
Supplement: sj-pdf-1-sjs-10.1177_1457496920976053 – Supplemental material for Computed Tomography Verified Prevalence of Incisional Hernia 1 Year Postoperatively after Colorectal Cancer Resection [file sj-pdf-1-sjs-10.1177_1457496920976053.pdf]

Supplement 1.

Summary of the patients included in the study and the ones excluded due to missing follow-up CT. The measure is either the median (quartile 1 and 3), the percentage (n/N indicating the fraction) or the mean (standard deviation) and number of observations. Unless otherwise stated, p-values were calculated using Pearson's χ^2-test. † indicates Student's T-test, ‡ Person's χ^2-test on grouped data and ⸸ Mann-Whitney U test. For surgical technique, non-converted and converted minimally invasive surgery, were combined when calculation thee p-value (*). Significance levels used is 0.05 and significant results are marked in bold.

Variable		Included median (Q_1, Q_3)	Included % (n/N) or mean (SD) and N	Excluded due to missing CT median (Q_1, Q_3)	Excluded due to missing CT % (n/N) or mean (SD) and N	p-value
Age [years]		70 (63, 76)	68.8 (10.8), 1231	78 (67, 84)	74.7 (12.5), 351	**<0.001**†
Gender	male		53.3 (656/1231)		50.7 (178/351)	0.394
	female		46.7 (575/1238)		49.3 (173/351)	
BMI [kg/m^2]		25.3 (22.8, 28.1)	25.8 (4.9), 1229	24.5 (22.2, 27.4)	24.9 (4.2), 315	0.001†
Resected area	colon		62.4 (767/1230)		77.7 (272/350)	**<0.001**
	rectum		37.6 (463/1230)		22.3 (78/350)	
ASA-class	1		14.6 (178/1222)		5.8 (20/344)	**<0.001**⸸ **<0.001**‡
	2		51.3 (627/1222)		38.1 (131/344)	
	3		33.2 (406/1222)		49.7 (171/344)	
	4		0.9 (11/1222)		6.1 (21/344)	
	5		0.0 (0/1222)		0.3 (1/344)	
Operation type	elective		88.9 (1092/1228)		74.9 (262/350)	**<0.001**
	acute		11.6 (142/1228)		25.1 (88/350)	
Bleeding [ml]		250 (100, 500)	1169	300 (100, 500)	323	0.340†
Operation time [minutes]		232 (175, 336)	1210	209 (157, 313)	347	0.001†
T-stage	T0		1.5 (19/1226)		1.4 (5/350)	**0.015**⸸ 0.303‡
	T1		3.7 (45/1226)		10.3 (36/350)	
	T2		13.2 (162/1226)		9.1 (32/350)	
	T3		54.5 (668/1226)		39.1 (137/350)	
	T4		26.9 (330/1226)		39.7 (139/350)	
	TX		0.2 (2/1226)		0.3 (1/350)	
N-stage	N0		51.3 (630/1228)		46.9 (164/350)	**0.030**⸸ 0.150‡
	N1		31.4 (386/1228)		29.1 (102/350)	
	N2		16.6 (204/1228)		23.1 (81/350)	
	NX		0.7 (8/1228)		0.9 (3/350)	
Neoadjuvant therapy			13.4 (165/1228)		10.0 (35/350)	0.088
Preoperative radiation			26.0 (319/1228)		12.9 (45/350)	**<0.001**
Stoma			45.0 (549/1221)		44.5 (155/348)	0.889
Wound infection			5.4 (66/1231)		6.6 (23/351)	0.393
Wound rupture			2.6 (32/1231)		4.0 (14/351)	0.172
Reoperation			1.6 (20/1231)		2.8 (10/351)	0.138
Incisional hernia			25.9 (319/1231)		-	
Procedure	ileocecal resection		0.4 (5/1229)		2.3 (8/350)	
	right hemicolectomy		31.7 (390/1229)		36.3 (127/350)	
	transverse colectomy		1.1 (13/1229)		2.6 (9/350)	
	left hemicolectomy		5.9 (73/1229)		6.9 (24/350)	
	sigmoid colectomy		14.1 (173/1229)		16.0 (56/350)	
	colectomy		2.3 (28/1229)		4.3 (15/350)	
	anterior resection		16.9 (208/1229)		6.9 (24/350)	
	abdominoperineal resection		17.5 (215/1229)		10.3 (36/350)	
	Hartmann's operation		10.1 (124/1229)		14.3 (50/350)	
	appendectomy		0.0 (0/1229)		0.3 (1/350)	
Surgery technique	open surgery		83.5 (1028/1231)		87.2 (306/351)	0.095
	non-converted laparoscopy		13.2 (162/1231)		12.8 (45/351)*	
	converted laparoscopy		3.3 (41/1231)			
IH surgery			3.7 (45/1231)			
IH surgery technique	JAD10, suture		0.2 (2/1231)			
	JAD20, onlay		0.2 (3/1231)			
	JAD30, inlay		0.5 (6/1231)			
	JAD33, inlay+relieving incision		0.1 (1/1231)			
	JAD40, sublay		1.9 (24/1231)			
	JAD43, sublay+relieving incision		0.1 (1/1231)			
	JAD70, multiple layers		0.2 (2/1231)			
	not specified		0.4 (5/1231)			
	multiple		0.1 (1/1231)			
